# Bronchiolitis in Children ‒ Do We Choose Wisely?

**DOI:** 10.34763/devperiodmed.20182204.323328

**Published:** 2019-01-14

**Authors:** Beata Kusak, Emilia Grzesik, Kaja Konarska-Gabryś, Zofia Pacek, Barnaba Piwowarczyk, Grzegorz Lis

**Affiliations:** 1 Department of Pediatrics, Chair of Pediatrics, Jagiellonian University Medical College, Krakow, Poland

**Keywords:** bronchiolitis, benchmark, quality of care, zapalenie oskrzelików, punkty kontroli, jakość opieki

## Abstract

**Aim:**

*The aim of our study was to evaluate the adherence to the 2014 American Academy of Pediatrics guidelines for bronchiolitis*.

**Material and methods:**

*The study measured the utility of diagnostic and therapeutic procedures in children with the first episode of bronchiolitis in their lives hospitalized at the University Children’s Hospital in Krakow, Poland, between September 2014 and March 2015. The results were compared with the achievable benchmarks of care (ABCs) for hospitalized children with bronchiolitis. Hospital performance was measured by five clinical indicators: chest X-ray utilization, viral testing implementation, steroids, antibiotics and bronchodilator prescriptions. Odds ratios (OR) with 95% confidence intervals (95%CI) were calculated for comparisons between hospital performance and ABCs*.

**Results:**

*There were 214 children in the study group (56% were boys). Chest X-ray was performed in 95% of the children, while ABCs indicate 32.4% (OR=42; 95%CI [30, 31, 32, 33, 34, 35, 36, 37, 38, 39, 40, 41, 42, 43, 44, 45, 46, 47, 48, 49, 50, 51, 52, 53, 54, 55, 56, 57, 58]); viral testing in 67.9% children, whereas ABCs indicate 0.6% (OR=350; 95%CI [155-790]). Steroids were prescribed in 62% of the patients (ABCs=6.4%; OR=24; 95 %CI [18, 19, 20, 21, 22, 23, 24, 25, 26, 27, 28, 29, 30, 31]), similarly antibiotics in 62% (ABCs=18.5%; OR=20; 95 %CI [1525]), bronchodilators were administered in 86% patients (ABCs=18.9%; OR=27; 95%CI [21, 22, 23, 24, 25, 26, 27, 28, 29, 30, 31, 32, 33, 34]). All the analyzed procedures were used dozens of times more often than suggested by ABCs (the difference is highly statistically significant)*

**Conclusions:**

*Overuse of ineffective procedures and therapies in bronchiolitis remains common, with overuse of chest X-rays, viral testing, prescriptions of bronchodilators, corticosteroids and antibiotics. We should focus our efforts on strategies aimed at decreasing the procedures that have no benefit for children with bronchiolitis*.

## Streszczenie

**Cel:**

*Celem pracy była ocena stopnia zastosowania wytycznych Amerykańskiej Akademii Pediatrii z 2014 r. dotyczących postępowania w zapaleniu oskrzelików (ZO) u dzieci*.

**Materiał i metody:**

*Oceniono zakres zastosowania procedur diagnostyczno-terapeutycznych u dzieci z pierwszym w życiu epizodem ZO hospitalizowanych w okresie wrz. 2014 – mar. 2015 w Uniwersyteckim Szpitalu Dziecięcym w Krakowie. Oceniono wykonywanie radiogramów klatki piersiowej i wymazu w kierunku wirusów oraz zastosowanie steroidów systemowych, antybiotyków i nebulizacji z beta2--mimetyków. Obserwowane częstości danych procedur porównano z punktami referencyjnymi (benchmarkami), określającymi zalecany poziom jakości opieki nad dziećmi z ZO. Wyliczany iloraz szans (IS) oraz jego 95% przedział ufności (95%PU) wskazuje na krotność przekroczenia i istotność różnic od danych benchmarków*.

**Wyniki:**

*Grupę badawczą stanowiło 214 (56% chłopców) dzieci. Radiogramy klatki piersiowej wykonano u 95% dzieci, natomiast benchmark zaleca częstość równą 32%, z tego wyznaczono IS=42, 95 %PU [3058]. Wymaz w kierunku wirusów wykonano u 67,9% (benchmark=0,6%; IS=350, 95%PU [155-790]). Leczenie steroidami systemowymi zastosowano u 62% pacjentów (benchmark=6%; IS=24, 95%PU [1831]), również u tej samej częstości (62%) stosowano leczenie antybiotykiem (benchmark=19%; IS=20, 95%PU [15, 16, 17, 18, 19, 20, 21, 22, 23, 24, 25]), u 86% pacjentów podawano nebulizacje z beta2-mimetyków (benchmark=19%; IS=27, 95%PU [21, 22, 23, 24, 25, 26, 27, 28, 29, 30, 31, 32, 33, 34])*.

**Wnioski:**

*Zastosowanie procedur diagnostyczno-terapeutycznych w grupie dzieci hospitalizowanych z powodu pierwszorazowego epizodu ZO jest znacząco częstsze niż wyznaczane przez dostępne punkty odniesienia (benchmarki) i nadużywane w świetle dostępnych zaleceń. Powinniśmy skoncentrować nasze działania na zmniejszenie wykonywania nieskutecznych procedur*.

## Introduction

Bronchiolitis is one of the most common causes of emergency visits and hospital admissions among infants and young preschool children. In 2014 the American Academy of Pediatrics (AAP) published guidelines to standardize and improve the quality of care for children with bronchiolitis [1]. Those clinical practice guidelines indicate diagnostic and therapeutic procedures whose effectiveness was proven in controlled clinical trials. They are directed at improving the clinical outcome as well as the safety of the hospitalized children with bronchiolitis. In common practice, evidence-based recommendations discourage the routine use of viral testing, chest X-rays or treatment with antibiotics, steroids and bronchodilators. The guidelines serve as recommendations and not an obligation to follow specific action in every patient, leaving the final decision to the judgement of the clinician. However, these guidelines and others support the “Choosing Wisely” campaign. The initiative that seeks to advance a national dialogue on avoiding unnecessary medical tests, treatment and procedures.

The aim of our study was to evaluate the adherence to the 2014 AAP guidelines [1]. We measured the utility of diagnostic and therapeutic procedures in children suffering the first episode of bronchiolitis in their lives and hospitalized in the University Children’s Hospital in Krakow, Poland between September 2014 and March 2015. We compared our results with achievable benchmarks of care (ABCs) for hospitalized children with bronchiolitis [2]. ABCs indicate average resource utilization among the high-performers and can serve as a comparison to the local level of inpatient care.

We analyzed those data as an initial phase of a study focused on improving the care of children with bronchiolitis in our hospital. The next phases will be developing educational tutorials summarizing AAP guidelines and structured daily charts with the respiratory scoring for hospitalized children. We presume that these instruments will improve the implementation of APP recommendations in our hospital and in this way improve the level of patient care.

## Material and methods

**Fig. 1:**
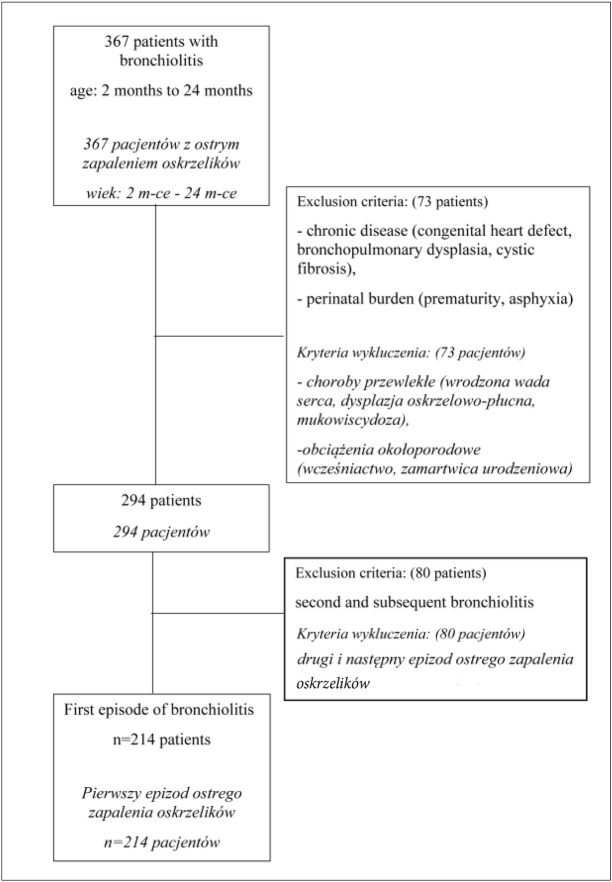
Study flow chart. *Ryc. 1. Schemat badania*.

The hospital database was searched, admissions to the Intensive Care Unit were omitted. Children aged 2 to 24 months with a discharge diagnosis of bronchiolitis were included. The exclusion criteria were as follows: chronic diseases (i.e. congenital heart defect, bronchopulmonary dysplasia, cystic fibrosis), perinatal burdens (i.e. prematurity, asphyxia), second and subsequent bronchiolitis. Out of the 367 children identified across the database, 214 (56% boys) children were included for further analysis (see Fig. 1).

Hospital performance was measured by five clinical indicators: chest X-ray utilization, viral testing implementation, steroids, antibiotics and bronchodilator prescriptions. The odds ratios (OR) with 95% confidence intervals (95% CI) were calculated for comparisons between our hospital performance and ABCs. An odds ratio is a relative measure of effect, which allows the comparison of the study group (our hospital) relative to the reference group (ABCs). The OR represents the odds of these procedures (clinical indicators) occurring in our hospital, compared to the odds of the procedures (clinical indicators) occurring in others hospitals where ABCs were implemented. The value of OR>1 means that the procedure (clinical indicator) was more likely to occur in our patients’ group than in the reference group (ABCs).

## Results

The median age of the study group was 5 months [inter quartile range IQR: 2.4-10.1]. The most common presenting symptoms were: rhinorrhea (87.7%), tachypnoea (65.7%) and wheezing (60.2%). Reduced oxygen saturation (SpO2 <90%) was revealed in 18.7% children on the admission day, and 21% the children were on supplemental oxygen during hospitalization. The median length of stay was 9 days [IQR: 7-11]. Moreover, there were identified nosocomial infections (mostly diarrhea) in 49% of the study children.

The results of our hospital’s performance (measured by the frequency of procedure utilization) are shown in Table 1. It has been noticed that all the analyzed procedures were used a dozens of times more often than suggested by ABCs (a highly statistically significant difference) (Tab. I).

## Discussion

The evidence-based guidelines for bronchiolitis are widely known among pediatricians, however, as our study showed, not so widely used. Several factors may contribute to such a practice. The AAP guidelines do not indicate any effective pharmacological treatment and list ineffective procedures, emphasizing instead supportive care with oxygen supplementation and proper hydration. Moreover, all the clinician should do is vigilant watching. This approach is consistent with the campaign “Choosing Wisely” [3]. The concept of this initiative is reducing unnecessary tests and procedures, not only to lower health care costs, but most of all to improve the patient’s safety and care.

Macias et al. showed in their study that diagnostic test utilization and treatment interventions are not attributable to clinical patients’ characteristics – fever, severity of illness, but presumably represent institutional factors [4]. Doctors request tests for their patient simply because they are accessible. Moreover, Jeffrey et al. demonstrated that readmission within 4 weeks of initial discharge of children hospitalized due to bronchiolitis was not associated with medical management or any modifiable factor [5]. We do not risk our patients’ safety when withdrawing some inefficient tests and procedures.

The key to making decisions wisely is by working out a reliable evaluation of a patient with bronchiolitis. Many pediatricians tend to rank their patients as being in a worse condition than the “average” child with bronchiolitis, which justifies their overuse of the diagnostic and therapeutic actions. In such situations, e.g. if crackles are found, bacterial pneumonia is diagnosed and an antibiotic is administered. If a wheeze is prominent, early asthma is suspected and steroids are administered [6]. To avoid such overestimations, the AAP recommends using objective measures of respiratory status, especially when judging response to interventions. However, so far, we have no ideal tool for ranking children with bronchiolitis. Here are some examples. Respiratory Distress Assessment Instrument (RDAI) and Respiratory Assessment Change Score (RACS) are the most frequently used measurement instruments in bronchiolitis clinical trials[7, 8]. They take into account such features as: the presence of wheezing (expiratory, inspiratory and their location), the use of accessory muscles (chest retractions) and the respiratory rate. The modified Wood’s Clinical Asthma Score (M-WCAS) is a validated tool for bronchiolitis [9]. It takes into account: oxygen saturation, inspiratory breath sounds, expiratory wheezing, use of accessory muscles and mental status. Those clinical scoring systems may be helpful when evaluating severity in children with bronchiolitis.

### Benchmarks vs hospital performance

The AAP guidelines recommend that routine chest X-rays should not be obtained, because it does not affect the patient’s outcome and on the contrary, can increase antibiotic use and extend the length of hospital stay. However, the ABCs showed that even in top pediatric hospitals, 32.4% of the children with bronchiolitis need chest X-rays. Those are probably children with additional indications for this procedure, like toxic appearance, prominent tachypnea or substantial respiratory effort, reduced oxygen saturation or just clinical judgement [10]. In our study almost all the children (95.3%) have chest X-rays, which seems to indicate that chest X-ray is used more as part of child’s routine examination than making an honest decision. Because of the retrospective nature of our study we cannot evaluate the severity of the patient’s condition and true indication for this procedure. We can only speculate that in at least 21% children (who were on oxygen therapy), the usefulness of chest X-ray would be justified.

The AAP guidelines recommend that viral testing should not be obtained routinely. The ABCs showed that common hospital practice is close to this recommendation (the estimated benchmark for viral testing was 0.6%). In our study viral testing was done in over half of the children (67.9%). The high use of this procedure may be justified by the willingness to cohort our patients. Moreover, Doan et al. showed that there is a trend towards prescribing fewer antibiotics when rapid viral tests are performed, especially in emergency departments, but this finding was not statistically significant. However, they found that rapid viral testing reduces the use of chest X-rays[11].

The AAP guidelines recommend that antibacterial medications should not be administered unless there is a concomitant bacterial infection, or a strong suspicion of one. The ABCs assess such a need in 18.5 % of the children with bronchiolitis. In our study 62.1% children were treated with antibiotics (i.e. 20-fold more often than the ABCs). However, we were not able to differentiate between early and postponed antibiotic administration.

Several studies showed that bacterial super-infection in children with bronchiolitis is rather low. The prevalence of bacteremia in those children is around 0.6 – 1.1% when using conventional blood culture and up to 10% when using molecular methods (PCR) [12]. Bacterial super-infection should be considered in the most severe cases treated with oxygen supplementation and/or mechanical ventilation. Fever alone is not a good predictor of bacteremia.

The AAP guidelines recommend that neither systemic corticosteroids nor bronchodilators should be administered in any setting. The calculated ABCs for steroid use was 6.4%, but there was wide variation between hospitals (median use was almost triple the benchmark), while the ABCs for bronchodilators was 18.9%. In our study 62.1% children were treated with steroids (i.e. 24-fold more than benchmark) and 86.4% children were treated with bronchodilators (i.e. 27-fold more than the benchmark). We did not differentiate one dosage of steroids/bronchodilators and a prolonged course of steroids/bronchodilators. The high usage of steroids and bronchodilators is probably due to clinical similarities between bronchiolitis and asthma exacerbation. However, we cannot forget that the pathophysiology of virus-induced wheezing is different from the wheeze in asthma. The high score for bronchodilator usage can be explained by previous AAP guidelines indicating assessment of bronchodilator response before ordering regular treatment with beta-agonist [13].

It turns out that the hardest decision for pediatricians is to restrict their interventions, even though there are many proven data which show that a higher utilization of a procedure was associated with increased length of hospital stay, without the benefit of decreased readmissions [14, 15].

**Tabela I:** – Porównanie wyników uzyskanych w badaniu z benchmarkami (ABCs). Table I. Comparison of the hospital performance in this study and achievable benchmarks of care (ABCs).

Procedure *Procedura*	Hospital performance Wynik uzyskany w badaniu	ABCs *ABCs*	OR [95% CI]* IS [95% PU]*
Chest X-ray Rtg klatki piersiowej	95.3%	32.4%	42.3 [30.7-58.3]
Viral test Wymaz w kierunku wirusów	67.9%	0.6%	350.4 [155.3-790.5]
Steroid prescriptions *Użycie steroidów*	62.1%	6.4%	24.0 [18 – 31.8]
Antibiotic prescriptions *Użycie antybiotyków*	62.1%	18.5%	20.1 [15.7 – 25.7]
Bronchodilator prescriptions *Użycie* *beta-2-mimetyków*	86.4%	18.9%	27.3 [21.4 – 34.7]
* p<0,0001 for all variables OR – odds ratio; 95%CI - 95% confidence interval *p<0,0001 dla wszystkich zmiennych *IS – iloraz szans; 95%PU - 95% przedział ufności*			

Instead of clarifying our high procedure utilization in children with bronchiolitis, we should undertake some actions to improve the situation. Several studies have showed that APP guideline implementation has improved in time [16] and further improvement can be expected if educational tutorial interventions are performed [17, 18]. The more multidirectional intervention the better the outcome that can be obtained. Not only educational lectures (conventional ones or webinars) or printed guidelines/ clinical pathway/ point-of-care algorithm are important, but relationships in medical teams are also crucial [19]. Some interventions work better when tailored to a certain group. For this, efforts to reduce radiographs should be focused on clinicians in emergency and outpatient departments because chest X-rays are generally obtained in these places [10]. Also improvement of patients’ evaluation by respiratory scoring is significantly related to decreasing bronchodilator usage [18].

## Conclussion

Overuse of ineffective procedures and therapies in bronchiolitis remains common, with overuse of chest X-rays, viral testing, prescriptions of bronchodilators, corticosteroids and antibiotics. According to state-of the-art data, our current efforts should be focused on strategies aimed at decreasing procedures that have no benefit for children with bronchiolitis.
